# Medical News from India

**Published:** 1895-01-26

**Authors:** Ernest Hart


### Medical News from India.

(Communicated by Mrs. Ernest Hart.)

Contemporaneous with the Medical Congress, two movements are being set on foot which we trust will be fruitful of the most beneficial results as to the prevention of disease and suffering and the saving of human life in India. One is to induce the Mohammedans of India to themselves take the initial and necessary steps to render the pilgrimage to Mecca no longer the means of disseminating and propagating cholera, and the other to found in the cities of India branches of the National Health Society to diffuse the knowledge of practical sanitation among the people, and more particularly among the women of India. In support of the former, Mr. Ernest Hart was invited by the orthodox Mohammedans to address them on the means of preventing cholera at Mecca and among the pilgrims to the holy shrine, and at the meeting there was a very large attendance of orthodox Mohammedans.

Mr. Hart pleaded that the Mohammedans themselves should use their influence and should make a collective representation to the Shereef of Mecca, the Barbar, and especially to the head of Islam, the Sultan, to prevent the great loss of life now incurred. Mr. Hart concluded by sketching out a practical scheme of operation, and making a number of suggestions. The audience was distinctly sympathetic, and frequently interrupted with bursts of genuine applause.

A most influential committee of orthodox Mohammedans has since been formed to carry out Mr. Hart's suggestions. It is sincerely to be hoped that this committee will do effective work, and that one source of danger to humanity may be removed, and the sufferings of the Mohammedan pilgrims enormously diminished. The suggestion to form branches of the National Health Society in India has been taken up with much warmth and sincerity.

